# The Complexity of the Root Canal Anatomy and Its Influence on Root Canal Debridement in the Apical Region: A Review

**DOI:** 10.7759/cureus.49024

**Published:** 2023-11-18

**Authors:** Rosnani Mamat, Nik Rozainah Nik Abdul Ghani

**Affiliations:** 1 Conservative Dentistry and Endodontics, School of Dental Sciences, Health Campus, Universiti Sains Malaysia, Kota Bharu, MYS

**Keywords:** apical canal, long oval-shaped, oval-shaped, debridement, root canal systems

## Abstract

The main goal of root canal treatment is to eliminate the infection in the complex root canal system for the long-term preservation of a functional tooth. Proper debridement of the root canal system, especially in the apical portion, is essential for successful root canal treatment. The complexity of the canal anatomy in the apical region plays a crucial role in reducing the microbial load. Therefore, clinicians must have a thorough knowledge of the anatomy of the root canal system and its variations, especially in the apical portion. Root canal configurations in cross-section have been classified as round, oval, long oval, flattened, or irregularly shaped. Treating oval, long oval, flattened, or irregularly shaped canals is challenging and should be approached differently than a circular canal. Recognizing the root canal shape and apical anatomy determines the different strategies to be used in cleaning, shaping, and obturation to achieve the best result of root canal treatment. The recent development of the instrumentation system improves the treatment outcome for clinicians and patients. This review aimed to discuss the definition, prevalence, and instrumentation for cleaning and shaping in the apical area with the complexity of root canal systems. Therefore, with the aid of this review, we can better understand the variations in the anatomy of the root canal, especially at the apical portion.

## Introduction and background

Root canal treatment (RCT) aims to repair and save a tooth that is severely decayed or infected [1]. The infected pulp tissue, including its nerve and blood supply, is removed during RCT treatment. During RCT, three stages or steps are performed: Access cavity, root canal preparation (biomechanical preparation and chemomechanical debridement), and obturation [2]. The elimination of bacteria from the root canal can be achieved through thorough chemomechanical debridement and instrumentation of the canal, particularly in the apical part. This is essential for the long-term success of RCT [3]. One of the primary objectives is to clean and shape the canal to a recommended diameter, enabling the placement of a homogeneous root canal filling. The anatomy of the root canal system is complex and varies in shape, making it difficult to remove organic tissue and reduce the microbial load, especially in the apical portion [4]. This complexity can have a significant impact on the debridement process and the overall success of the RCT. Therefore, appropriate root canal preparation procedures are required for instrumentation, especially in the apical portion of the root canal, as the apical area is often irregularly shaped, has various ramifications, and is usually not configured all around [5]. In addition, this area is preferentially used as a niche for the remaining microorganisms. The accumulated bacteria in this complex anatomical area can lead to reinfection if not adequately removed. The apical portion of a root canal is the narrowest part and represents the last region in the root canal system [5]. Instrumentation and root filling in this area have been deemed challenging. Ricucci D and Langeland K [6] reported that material extruded beyond the apical constriction may promote inflammation and a foreign body reaction. Therefore, precise instrumentation in the apical portion is considered a crucial step in root canal preparation [2]. Although biomechanical instruments have a variety of sizes and tapers, they are still insufficient to adequately clean the root canal system [7]. This statement is supported by most previous studies showing that mechanical instruments do not make full contact with all root canal walls [6-11]. This finding was more pronounced in canals with shapes other than round, owing to the design of the instruments, which may not correspond to the natural anatomy of the root canal. Versiani MA et al. have shown that the root canal's shape can influence the instrumentation outcome [10]. Therefore, comprehensive knowledge and understanding of the root canal anatomy are required to achieve RCT success. The purpose of this review is to highlight the different cross-sectional shapes of root canals in the apical portion and discuss the different aspects of instrumentation that can be considered for debridement in this area.

## Review

Definition of root canal anatomy

According to Jou YT et al., cross-sectional root canal configurations have been classified based on the root canal anatomy into several shapes: round (circular), oval, long oval, flattened (flat, ribbon), and irregularly shaped canals [12]. These shapes largely depend on the ratio of the buccolingual and mesiodistal diameters, as illustrated in Table 1.

**Table 1:** Classification of cross-sections of the root canal system.

No.	Shape of canal	Description
1	Round	Buccolingual diameter equal to or less than mesiodistal diameter.
2	Oval	Buccolingual diameter greater than mesiodistal diameter (up to two times more).
3	Long Oval	Buccolingual diameter two or more times greater than mesiodistal diameter (up to four times more).
4	Flattened	Buccolingual diameter four or more times greater than mesiodistal diameter.
5	Irregular	Cannot be defined by categories 1–4.

Prevalence

Numerous studies have been conducted to evaluate the cross-sectional shape of the canal in the apical portion, as detailed in Tables 2-4. Most of these studies focused on the canal's shape within 1 mm to 5 mm from the apex. The shapes were categorized as round (Table 2), oval (Table 3), and long-oval (Table 4), with each table providing detailed information on the methods used and the number of teeth examined. The findings varied according to the position of the teeth. In these studies, sections were typically made using a low-speed saw, and the images were then measured under a microscope at magnifications of either 30× or 50× [4,13]. In contrast, Kacharaju KR et al. utilized X-ray films in two directions (buccolingual and mesiodistal) [14]. Additionally, they employed three-dimensional imaging techniques, either cone beam computed tomography (CBCT) or micro-computed tomography (micro-CT), to categorize the apical shape of the canal [15-19]. The prevalence of each canal shape in the apical portion, according to tooth position, is summarized in Table 2 (round), Table 3 (oval), and Table 4 (long-oval). Based on previous studies [4,15,18-19], it can be concluded that central and lateral incisors exhibit varying canal shapes, including round, oval, and long oval. Most canines have an oval-shaped canal, with a prevalence of approximately 96% [18], and 6-11% exhibit a long-oval shape [4]. In premolars, the prevalence of oval canals is about 37%, and long-oval-shaped canals range from 7-63% [4,14]. Meanwhile, both maxillary and mandibular molars predominantly show oval to long-oval-shaped canals, with a prevalence of ≥10%.

**Table 2:** Percentage of round canals (≤1) at 1-5 mm from the apex. CBCT: Cone-beam computed tomography; CCD: Charge-coupled device; MD: Mesiodistal; BL: Buccolingual; Micro-CT: Micro-computed tomography.

Tooth (canal) position		Number of teeth	Number of cross sections	Prevalence	Method	Authors
Central Incisor	Maxillary	378	378	Not mention	CBCT scan at 110kV using I-CAT viewer software	Razumova S et al. [18]
	Mandibular	1472	842	96.2%	CBCT scan at 85kV using OnDemand3D software	Shemesh A et al. [19]
		100	300	12.3%	Sectioned with low-speed saw, imaged with high resolution CCD camera at 50x magnification and measured with UT ImageTool version 1.21.	Mauger MJ et al. [13]
Lateral Incisor	Maxillary	392	392	Not mention	CBCT san with voltage 110kV and used I-CAT viewer software	Razumova S et al. [18]
	Mandibular	1508	902	95.8%	CBCT scan with voltage 85kV and used OnDemand3D software	Shemesh A et al. [19]
Premolar	Maxillary	310	310	Not mention	CBCT san with voltage 110kV and used I-CAT viewer software	Razumova S et al. [18]
		44	57	1.7%	Micro-CT with voltage 130 kV and 50 µm isotropic resolution	Arfianti RP et al. [16]
	Mandibular	100	37	20%	Radiographic film in mesiodistal (MD) and buccolingual (BL) direction	Kacharaju KR et al. [14]
		347	347	Not mention	CBCT san with voltage 110kV and used I-CAT viewer software	Razumova S et al. [18]
Molar DB canal	Maxillary	250	250	Not mention	CBCT san with voltage 110kV and used I-CAT viewer software	Razumova S et al. [18]
Molar Palatal canal	Maxillary	250	250	Not mention	CBCT scan with voltage 110 kV and used I-CAT viewer software	Razumova S et al. [18]
Molar Distal canal	Mandibular	100	100	3.8-8.9%	Micro-CT with voltage 50 kV and 19.6 µm isotropic resolution	Filpo-Perez C et al. [17]

**Table 3:** Percentage of oval canals (>1 up to 2) at 1-5 mm from the apex. CBCT: Cone beam computed tomography; Micro-CT: Micro-computed tomography; MD: Mesiodistal; BL: Buccolingual; MB: Mesio-buccal; DB: Disto-buccal; ML: Meso-lingual.

Tooth (canal) position		Number of teeth	Number of cross sections	Prevalence	Method	Authors
Central Incisor	Maxillary	300	300	Not mentioned	CBCT scan at 110kV using I-CAT viewer software	Razumova S et al. [18]
	Mandibular	19	95	47%	Horizontal sectioning and microscope measurement at 30x magnification	Wu MK et al. [4]
		1472	4	0.5%	CBCT scan with voltage 85kV and used OnDemand3D software	Shemesh A et al. [19]
		481	481	95%	CBCT scan with voltage 110kV and used I-CAT viewer software	Razumova S et al. [18]
		340	257	68-83%	Micro-CT with voltage 50kV and 19.6 µm isotropic resolution	Milanezi de Almeida M et al. [15]
Lateral incisor	Mandibular	1508	9	0.95%	CBCT scan with voltage 85kV and used OnDemand3D software	Shemesh A et al. [19]
		475	475	95%	CBCT scan with voltage 110kV and used I-CAT viewer software	Razumova S et al. [18]
		340	56	24-63%	Micro-CT with voltage 50kV and 19.6 µm isotropic resolution	Milanezi de Almeida M et al. [15]
Canine	Maxillary	357	357	Not mentioned	CBCT scan with voltage 110kV and used I-CAT viewer software	Razumova S et al. [18]
	Mandibular	456	456	96%	CBCT san with voltage 110kV and used I-CAT viewer software	Razumova S et al. [18]
Premolar	Maxillary	20	100	38%	Horizontal sectioned and measured with microscope (x30)	Wu MK et al. [4]
		44	57	66.7%	Micro-CT with voltage 130kV and 50µm isotropic resolution	Arfianti R et al. [16]
	Mandibular	300	300	Not mentioned	CBCT san with voltage 110kV and used I-CAT viewer software	Razumova S et al. [18]
		100	22	37%	Radiographic film in mesiodistal (MD) and buccolingual (BL) direction	Kacharaju KR et al. [14]
Molar MB canal	Maxillary	20	100	40%	Horizontal sectioned and measured with microscope (x30)	Wu MK et al. [4]
		17	56	68.2%	Micro-CT with voltage 130kV and 50µm isotropic resolution	Arfianti R et al. [16]
		250	250	Not mentioned	CBCT san with voltage 110kV and used I-CAT viewer software	Razumova S et al. [18]
	Mandibular	75	375	39%	Horizontal sectioned and measured with microscope (x30)	Wu MK et al. [4]
		19	43	36.8%	Micro-CT with voltage 130kV and 50µm isotropic resolution	Arfianti R et al. [16]
Molar DB canal	Maxillary	17	56	94.1%	Micro-CT with voltage 130kV and 50µm isotropic resolution	Arfianti R et al. [16]
Molar Palatal canal	Maxillary	17	56	100%	Micro-CT with voltage 130kV and 50µm isotropic resolution	Arfianti R et al. [16]
Molar ML canal	Mandibular	19	43	100%	Micro-CT with voltage 130kV and 50µm isotropic resolution	Arfianti R et al. [16]
Molar Distal canal	Mandibular	100	100	27-64.5%	Micro-CT with voltage 50kV and 19.6 µm isotropic resolution	Filpo-Perez C et al. [17]
		19	43	68.4%	Micro-CT with voltage 130kV and 50µm isotropic resolution	Arfianti R et al. [16]

**Table 4:** Percentage of long oval canals (≥2) at 1-5mm from the apex CBCT: Cone beam computed tomography; Micro-CT: Micro-computed tomography; MB: Mesio-buccal; DB: Disto-buccal; ML: Meso-lingual.

Tooth (canal) position		Number of teeth	Number of cross sections	Prevelance	Method	Authors
Central Incisor	Maxillary	40	200	5-35%	Horizontally sectioned and measured with microscope at 30x	Wu MK et al. [4]
	Mandibular	40	200	10-56%		
		1472	17	1.94%	CBCT scan at 85kV using OnDemand3D software	Shemesh A et al. [19]
		340	257	14-25%	Micro-CT with voltage 50kV and 19.6 µm isotropic resolution	Milanezi de Almeida M et al. [15]
Lateral incisor	Mandibular	1508	20	2.12%	CBCT scan with voltage 85kV and used OnDemand3D software	Shemesh A et al. [19]
		340	56	27-41%	Micro-CT with voltage 50kV and 19.6 µm isotropic resolution	Milanezi de Almeida M et al. [15]
Canine	Maxillary	20	100	5-6%	Horizontal sectioned and measured with microscope (x30)	Wu MK et al. [4]
	Mandibular	20	100	5-11%	Horizontal sectioned and measured with microscope (x30)	Wu MK et al. [4]
Premolar	Maxillary	40	200	7-63%	Horizontal sectioned and measured with microscope (x30)	Wu MK et al. [4]
		44	57	24.6%	Micro-CT with voltage 130kV and 50µm isotropic resolution	Arfianti RP et al. [16]
	Mandibular	40	200	13-40%	Horizontal sectioned and measured with microscope (x30)	Wu MK et al. [4]
Molar MB canal	Maxillary	60	300	13-80%	Horizontal sectioned and measured with microscope (x30)	Wu MK et al. [4]
		17	56	22.7%	Micro-CT with voltage 130kV and 50µm isotropic resolution	Arfianti RP et al. [16]
	Mandibular	40	200	20-92%	Horizontal sectioned and measured with microscope (x30)	Wu MK et al. [4]
		19	43	47.4%	Micro-CT with voltage 130kV and 50µm isotropic resolution	Arfianti RP et al. [16]
Molar DB canal	Maxillary	20	100	11-30%	Horizontal sectioned and measured with microscope (x30)	Wu MK et al. [4]
		17	56	5.9%	Micro-CT with voltage 130kV and 50µm isotropic resolution	Arfianti RP et al. [16]
Molar ML canal	Mandibular	20	100	10-25%	Horizontal sectioned and measured with microscope (x30)	Wu MK et al. [4]
Molar Palatal canal	Maxillary	20	100	10-24%	Horizontal sectioned and measured with microscope (x30)	Wu MK et al. [4]
Molar Distal canal	Mandibular	20	100	24-30%	Horizontal sectioned and measured with microscope (x30)	Wu MK et al. [4]
		100	100	24-54%	Micro-CT with voltage 50kV and 19.6 µm isotropic resolution	Filpo-Perez C et al. [17]
		19	43	21.1%	Micro-CT with voltage 130kV and 50µm isotropic resolution	Arfianti RP et al. [16]

Anatomy of the apical region

The apical portion of the root canal system is characterized by highly complex and variable anatomy, as it has different shapes ranging from round to long oval-shaped canals [4,13-15,17-19]. This has a great impact on the successful outcome of RCT. According to a histological study by Ricucci D and Langeland K [6], the apical constriction is located just before the apical foramen. It is the best area to complete the root canal instrumentation and obturation of the root canal. Previous studies [6,8,20] have shown that more than 60% of the apical foramen is not located at the radiographic apex, with distances varying according to the individual's age. Their results showed that the distance between the apical foramen and the radiographic apex was 0.48 mm and 0.6 mm in the young and elderly, respectively. Moreover, the authors [6,8,20] found that microorganisms preferentially accumulate in this area, which is a leading cause of apical periodontitis after treatment. This condition often resolves due to pathological changes such as root resorption or canal calcification [6]. This underscores the strong justification for maintaining apical patency in cases of pulp necrosis or infection.

The technique of mechanical instrumentation

One of the goals of root canal preparation is to remove the infected dentin and shape the canal with an appropriate geometry to facilitate obturation of the root canal system [2]. This process can be performed with manual or rotary instruments or a combination of both. Many different techniques have been developed to achieve the biological goals of eliminating microorganisms from the root canal system, removing pulp tissue that may support microbial growth, and avoiding pushing debris beyond the apical foramen while facilitating obturation placement [1]. In the past, the step-back technique was developed using ISO-standardized 0.02-taper stainless steel hand files [21]. This technique, as described by Mullaney TP [22], involves starting instrumentation at the apical portion of the root canal and then extending it coronally. However, the inherent inflexibility of stainless-steel files often leads to iatrogenic damage to the original canal shape, especially in curved canals, when using the step-back technique. Several new techniques, including the step-down technique, have been developed to address this issue. This technique starts with larger instruments at the canal opening and then works its way down to the apical region with smaller files [1]. Advanced technology in instrumentation has been developed to overcome the weaknesses of the various instrument systems available. Nickel-titanium (NiTi) technology was invented for the root canal treatment procedure to design and fabricate rotary endodontic instruments that are more effective than stainless steel instruments [23-24]. NiTi instruments have been developed for the fifth generation to improve the quality of chemomechanical debridement in the root canal system [24]. This development is shown in Table 5.

**Table 5:** Evolution of nickel-titanium files. NiTi: Nickel-Titanium; CM: Controlled memory; SAF: Self-adjusting file; EDM: Electrical discharge machining.

Generation	Characteristics	Type of Instruments
First	Introduced to the market during the mid-1990s. Featured passive cutting radial lands with fixed tapers of 0.04–0.06 over the full working lengths. Created smooth root canal walls, centered in the middle, and caused low procedural errors. However, it required numerous files to achieve instrumentation goals due to its complexity.	LightSpeed Endodontics (1992), Profile (Dentsply) (1993), Quantec (SybronEndo) (1996), GT system (Dentsply) (1998)
Second	Introduced in 2001. Featured active cutting edges with greater efficiency. Required fewer instruments for complete cleaning and shaping compared to the first generation. Provided fast preparation while preserving the original shape of root canals, even in curved and calcified cases. However, some degrees of canal transportation and a tendency for breakage during usage were noted.	ProTaper Universal (Dentsply), K3 (SybronEndo), Mtwo (VDW), Hero Shaper (Micro-Mega), I Race, and I Race Plus (FKG Dentaire)
Third	Introduced in late 2007. Manufacturers applied heating and cooling technologies to NiTi alloys to improve the safety of these instruments, especially in curved root canals. This resulted in reduced cyclic fatigue and a lower risk of instrument separation.	K3 XF Files (SybronEndo), Profile GTX Series (Dentsply), Controlled Memory (CM) Files HyFlex CM (Coltene), Vortex Blue (Dentsply Tulsa).
Fourth	Based on the reciprocation theory. Utilized a single file technique durng cleaning and shaping.	Wave One (Dentsply), Self-adjusting file (SAF) (ReDent Nova), Reciproc (VDW).
Fifth	Produces a mechanical wave of motion along the length of the NiTi file.	HyFlex/electrical discharge machining (EDM) (Coltene), Revo-S (Micro-Mega), One Shape (Micro-Mega), ProTaper Next (Dentsply).

Most current techniques for chemomechanical preparation use motor-driven NiTi alloy instruments, which have been shown to shape the canal with appropriate geometries and reduce the risk of major procedural errors [1,3]. To our knowledge, none of the instruments were ideal for preparing oval or long-oval root canals, as current motor-driven instruments allow for round preparations [3,7,9,25].

Apical instrumentation

According to the American Association of Endodontics (AAE) 2020 Glossary of Endodontic Terms, chemomechanical debridement is defined as the use of chemicals to irrigate the root canal, demineralize dentin, dissolve pulp tissue, and neutralize bacterial products and toxins. It is used in conjunction with biomechanical preparation [2]. Biomechanical preparation is defined as using rotary or reciprocating instruments and/or hand instruments to expose, clean, expand, and shape the pulp canal, usually in conjunction with irrigants. The use of a combination of both is mandatory in all cases of RCT, especially in complex root canal systems [1]. In endodontics, instruments have undergone several modifications in their design and heat treatments of alloys to improve the quality and predictability of root canal preparation [24]. For this reason, numerous studies [7,9-11,25-27] have been conducted to investigate the efficiency of advanced NiTi technology depending on the canal's shape, which is not round. According to Taha NA et al., who compared different instrument techniques such as Anatomic Endodontic Technology (AET), Hedstrom files, and EndoWave rotary NiTi files, the EndoWave NiTi file resulted in better cleanliness in the apical part of canals with an oval shape compared to AET and Hedstrom files [25]. In another study [7] by Guimarães LS et al., the TRUShape and reciprocal systems were compared to prepare oval canals. The results showed that both systems performed similarly, leaving approximately 20% of the area uninstrumented. This finding is corroborated by another study [10-11]. According to Zuolo ML et al., four instrumentation systems, such as BioRace, Reciproc, Self-Adjusting File (SAF), and TRUShape, were compared in oval canals [11]. The extent of untouched canal walls was not significantly different between the Reciproc, SAF, and TRUShape systems. Versiani MA et al. compared SAF, Reciproc, WaveOne, and ProTaper and found that none of the systems differed in the preparation of oval canals [10]. Therefore, the data from the different studies show that neither the technique nor the instruments can perform thorough debridement of oval or long oval canals.

Apical patency

Numerous studies [4,14,18,28] concluded that the apical portion is a crucial area in root canal preparation. During instrumentation, dentin debris may block the apical portion and cause ledge, apical transport, perforation, or difficulty reaching the corrected working length [5]. Therefore, the suggested method to avoid this problem is to use a patency file. According to the AAE 2020 Glossary of Endodontic Terms, apical patency was defined as a technique in which the apical portion of the canal is kept free of debris by recapitulation with a small file through the apical foramen. Recapitulation is reinserting small or fine files during canal preparation to keep the apical area clean and patent [2]. It is recommended to recapitulate with a size 10 k-file after each instrumentation prior to irrigation to avoid the accumulation and compaction of debris and tissue debris at the apical end of the root canal system [5]. However, the concept of apical patency is controversial. Some studies [29-30] have found that maintaining apical patency may increase postoperative pain or flare-ups. Shubham S et al. reported that pulp and preoperative dental pain influenced postoperative pain in the patency group [29]. Other studies [31-33] came to the opposite conclusion that maintaining apical patency leads to a better outcome because bacteria can be removed at the apical foramen. This finding is supported by Yaylali IE et al., who conducted a systematic review of randomized clinical trials (RCTs) involving 848 patients and found that maintaining apical patency did not increase postoperative pain in teeth with vital or non-vital pulp [33]. At the same time, no case of flare-up was reported. Furthermore, in an in vivo study of 80 endodontically treated teeth by Garg N et al., it was found that maintaining apical patency did not increase the incidence of postoperative pain [32]. In vivo studies by Vera J et al. [34-35] investigated the use of a radiopaque solution mixed with NaOCl in both small and large canals of premolars and molars. The results indicated that maintaining apical patency with a size 10 K-file during the cleaning and shaping stages significantly increased the delivery of irrigation solution to the apical third, even in smaller canals such as the mesial roots of lower molars, buccal roots of upper molars, and both roots of upper first premolars. It is important to note that NaOCl requires sufficient time and contact to dissolve organic tissue and affect microorganisms, especially when they are protected by biofilm [34-35]. Based on these findings, maintaining apical patency is recommended to optimize root canal debridement.

Apical size preparation

The question of how large the preparation should be to effectively disinfect the apical part of the root canal system is controversial in endodontics. Some authors [36-38] suggest a larger apical preparation than previously used to disinfect or clean the apical portion. A larger apical preparation allows better penetration of irrigants. However, others [39-40] argue that it removes unnecessary dentin walls and may weaken the root canal system. Lee OY et al.'s study of the oval and round apical root canal found that preparation size and irrigation technique have an impact on the cleanliness of the root canal system [37]. In this study, they prepared mandibular premolars with rotary NiTi of size 20 and 40 with a taper of 4%. Then, one group of specimens was irrigated with a syringe and needle, while ultrasonically activated irrigation was used for another group of specimens. Their study concluded that syringe and needle irrigation required greater preparation to achieve better debridement in the apical portion for both root canal shapes. For ultrasonically activated irrigation, the size of the preparation had no effect. However, the oval shape had more remaining pulp tissue and debris because the percentage of the untouched area was higher than for the round shape of the canal. Therefore, this study showed that the root canal's irrigation technique and shape influenced the root canal's debridement, especially in the apical portion. A study by Fatima S et al. also supported the results of the previous study [37] when they found that teeth were prepared with a file twice the size of the initial apical bandage file (IABF), with a conical file of 4% being inadequate compared to a conical file of 6% [36]. They measured postoperative pain for up to three days in 120 patients. Wu MK and Wesselink PR [27] reported that most teeth had canals with oval-round shapes, and the average taper of the original canal in the buccolingual direction was 0.10 mm/mm. In contrast, the taper in the mesiodistal direction was much less. They noted that only some teeth have a rounded canal that is parallel in both directions. They suggested that an ideal instrument taper is between 0.05 and 0.10 mm/mm diameter and that any taper less than 0.05 mm/mm would leave uninstrumented areas in the buccolingual orientation. However, to date, the minimum apical size of the preparation is still debated between clinicians and researchers.

## Conclusions

Although, to date, no instrument can provide the perfect result in mechanical instrumentation of the oval or long oval canal, a better result can be achieved if the right tools and instruments are used in the right specific cases. Therefore, clinicians must have a solid and comprehensive knowledge of the anatomy of the root canal, especially the apical part of the root, to ensure the best treatment for the patient. It is recommended that clinicians recognize the shape of the root canal based on preoperative radiographs before choosing the right instrumentation system and irrigation technique for the cleaning and shaping procedure.
